# Triple-orifice repair in severe barlow disease with multiple-jet regurgitation: report of mid-term experience

**DOI:** 10.1186/1749-8090-8-S1-O275

**Published:** 2013-09-11

**Authors:** C Fucci, G DeCicco, L Latini, G DiMatteo, G Coletti

**Affiliations:** 1 Cardiac Surgery Department, Civic Hospital, Brescia, Italy

## Background

Barlow disease represents a surgical challenge for mitral valve repair (MR) in the presence of mitral insufficiency (MI) with multiple regurgitant jets. We hereby present our mid-term experience using a midified edge-to-edge technique to address this peculiar MI.

## Methods

From March 2003 till December 2012, 28 patients (mean age 53.8+/- 6 years, 16 males) affected by severe Barlow disease with multiple jets were submitted to MR. Preoperative transesophageal echo (TEE) in all the cases showed at least 2 regurgitant jets, involving one or both leaflets in more than one segment. In all the patients, a triple-orifice valve (TOV) repair with annuloplasty was performed. Intraoperative TEE and postoperative transthoracic echocardiography (TT) were carried out to evaluate results of the TOV repair.

## Results

There was no in-hospital death and one late death (non-cardia related). At intraoperative TEE, the three orifices showed a mean total valve area of 2.98+/-0.3 cm2 (range: 2.5-3-3) with no residual regurgitation (2 cases of trivial) and no sign of valve stenosis (mean transvalvular gradient 4.5+/-1-3 mmHg.). At follow-up (mean: 68.9+/-12 months), TEE showed favourable MR and no recurrence of significant MI ( 6 cases of trivial and 1 of mild MI). Stress TEE was performed in 5 cases showing persistent effective velve function (2cases of trivial MI at peak exercise). All the patients showed significant NYHA functional class improvement.

## Conclusions

This report indicates that the TOV technique is effective in correcting complex Barlow mitral valves with multiple jets. Further studies are required to confirm long-term applicability and durability in more numerous cases.

